# Free Triiodothyronine Level Correlates with Myocardial Injury and Prognosis in Idiopathic Dilated Cardiomyopathy: Evidence from Cardiac MRI and SPECT/PET Imaging

**DOI:** 10.1038/srep39811

**Published:** 2016-12-22

**Authors:** Wenyao Wang, Haixia Guan, Wei Fang, Kuo Zhang, A. Martin Gerdes, Giorgio Iervasi, Yi-Da Tang

**Affiliations:** 1 Department of Cardiology, State Key Laboratory of Cardiovascular Disease, Fuwai Hospital, National Center for Cardiovascular Diseases, Chinese Academy of Medical Sciences and Peking Union Medical College, Beijing, China; 2 Department of Endocrinology and Metabolism, The First Affiliated Hospital of China Medical University, Shenyang, China; 3 Department of Nuclear Medicine, State Key Laboratory of Cardiovascular Disease, Fuwai Hospital, National Center for Cardiovascular Diseases, Chinese Academy of Medical Sciences and Peking Union Medical College, Beijing, China; 4 Department of Biomedical Sciences, New York Institute of Technology-College of Osteopathic Medicine, Old Westbury, New York, USA; 5 Clinical Physiology Institute, Consiglio Nazionale delle Ricerche (CNR), Pisa, Italy

## Abstract

Thyroid dysfunction is associated with poor prognosis in heart failure, but theories of mechanisms are mainly based on animal experiments, not on human level. We aimed to explore the relation between thyroid function and myocardial injuries in idiopathic dilated cardiomyopathy (IDCM) using cardiac magnetic resonance imaging (MRI), single-photon emission computed tomography (SPECT) and positron emission tomography (PET). Myocardial fibrosis was detected by late gadolinium enhancement (LGE) MRI, and myocardial perfusion/metabolism was evaluated by ^99m^Tc-MIBI SPECT /^18^F-FDG PET imaging. Across the quartiles of FT3, decreased percentage of segments with LGE and perfusion/metabolism abnormalities were found. As for FT4 and TSH levels, no significant distribution trend of myocardial injuries could be detected. In logistic analysis, FT3 was independently associated with the presence of LGE (OR: 0.140, 95% CI: 0.035–0.567), perfusion abnormalities (OR: 0.172, 95% CI: 0.040–0.738) and metabolism abnormalities (OR: 0.281, 95% CI: 0.081–0.971). After a median follow-up of 46 months, LGE-positive and FT3 < 2.77 pg/mL was identified as the strongest predictor of cardiac events (HR: 8.623, 95% CI: 3.626–16.438). Low FT3 level is associated with myocardial fibrosis and perfusion/metabolism abnormalities in patients with IDCM. The combination of FT3 level and LGE provides useful information for assessing the prognosis of IDCM.

With a growing body of evidence^1^,^2^,^3^, thyroid dysfunction has proven to be an important risk factor in the progression of cardiovascular diseases, although changes of thyroid status and their impact on prognosis have varied in particular populations. Our previous study^4^, together with other clinical evidence^5^,^6^, suggests that thyroid hormone (TH) levels correlate with cardiac function and hypothyroidism was a predictor of poor outcome in dilated cardiomyopathy (DCM). However, detailed understanding of mechanisms linking thyroid dysfunction and heart failure (HF) are warranted to support further studies on treatment.

Animal experiments have demonstrated that THs have complex effects on myocytes, matrix, vascular growth, and ventricular function through genomic or non-genomic mechanisms^7^,^8^,^9^. TH-induced vasodilation can occur within minutes by a rapid, non-genomic mechanism^10^. Both T3 and T4 have been shown to stimulate angiogenesis *in vitro*^11^,^12^. Our previous animal studies showed that hypothyroidism leads to impaired myocardial blood flow, associated with loss of small arterioles^7^. In hyperthyroidism, THs promote the breakdown of collagen by increasing matrix metalloproteinase-1 activity, leading to hypertrophy without increased fibrosis, at least in the earlier stages^13^. Conversely, increased accumulation of collagen can occur with low thyroid function, possibly via regulation of collagen gene expression through TH receptors^14^. A recent publication revealed anti-fibrotic effects of THs through miRNA signaling^15^. Nonetheless, the paucity of related human data leaves the question open whether low thyroid function is a beneficial or detrimental response in HF.

Clinically, application of cardiac magnetic resonance imaging (MRI) and radionuclide tests has become frequent in DCM patients for etiological diagnosis and risk stratification. Late gadolinium enhanced (LGE) MRI provides useful noninvasive evaluation of myocardial fibrosis. The presence of LGE has also been indicated as a predictor for long-term mortality^16^,^17^. Combining ^99m^Tc-MIBI single-photon emission computed tomography (SPECT) perfusion imaging and ^18^F-FDG positron emission tomography (PET) metabolic imaging enables the detection of ischemic and necrotic myocardium^18^. With myocardial fibrosis evaluated by cardiac MRI and myocardial perfusion/metabolism status measured by radionuclide tests, we explored the relationship between TH levels and different types of myocardial injuries in IDCM patients to provide direct mechanistic evidence and improve patient selection for management decisions.

## Methods

### Study Group

We conducted a prospective observational study of 71 IDCM patients who were admitted to Fuwai Hospital (National Center of Cardiovascular Diseases, China) from January 2010 to October 2011. This subgroup belongs to a cohort study of 458 IDCM patients previously reported^4^. The study disposition is showed in Supplementary Figure S1. The study protocol conformed to the ethical guidelines of the 1975 Declaration of Helsinki. The Institutional Review Board Committee (Fuwai Hospital) approved the study protocol. All patients enrolled in the study provided informed consent for MRI, PET/SPECT imaging and blood extraction. The diagnosis of IDCM was based on the WHO criteria^19^. Of note, significant coronary artery disease (50% diameter luminal stenosis in one or more coronary arteries) was ruled out by invasive coronary angiography for all patients in this study group. Exclusion criteria were as follow: history of severe valvular disease, hypertensive heart disease, tachycardia-induced cardiomyopathy, alcohol abuse, metal fragments in their bodies, implanted ferromagnetic devices or unsuitable to undergo nuclear imaging or MRI. Cardiac MRI, ^99m^Tc-MIBI myocardial perfusion SPECT imaging and ^18^F-FDG myocardial metabolic PET imaging were undertaken by all patients within an interval of 3–7 days.

### Thyroid function test and selection of cutoff value of thyroid hormone levels

Thyroid status was evaluated when HF symptoms could be controlled under regular oral medication before they were discharged, rather than in the acute phase. The median duration between hospital admission and thyroid function test was 10 days. Twelve-hour-fasting blood samples were drawn and the serum levels of TH and TSH were measured using radioimmunoassay (Immulite 2000; Siemens, Germany) in the Nuclear Medicine Department of Fuwai Hospital. The reference intervals of TH and TSH in our laboratory are as follows: TSH = 0.55–4.78 mIU/L; FT3 = 1.79–4.09 pg/mL; FT4 = 0.8–1.88 ng/dL; TT3 = 0.65–1.91 ng/mL; TT4 = 4.29–12.47 ug/dL. Distribution trend of myocardial injuries was explored across FT3 quartiles. In Cox survival analysis and Kaplan-Meier plot, we used the median value of FT3 (2.77) as cut-off for two reasons: first, we did comparison of segments and logistic analysis based on FT3 quartiles, so as sequential procedure, the prognosis analysis was also performed with previous cutoff; second, with only 71 IDCM patients, the strength of our current study is the complete information of cardiac MRI and radionuclide tests, while the small population limited the use of ROC curve to choose an accurate cutoff value, and this question might be further explored in a large population.

### Cardiac MRI and ^99m^Tc-MIBI SPECT/^18^F-FDG PET imaging

**Figure 1:**
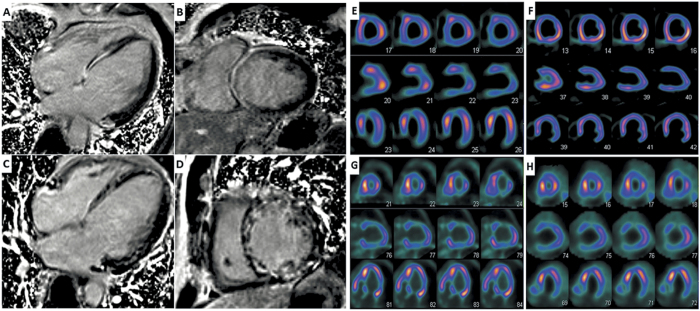
Representative examples of LGE-positive and perfusion/metabolism abnormality images. The typical LGE pattern in DCM is mid-wall enhancement in the interventricular septum (**A**,**B**). A diffuse pattern was observed in 5 out of 31 LGE-positive patients (**C**,**D**). Severely reduced uptake of ^99m^Tc-MIBI in apex, anterior and inferior wall (**E**) while ^18^F-FDG PET imaging showed normal FDG uptake (**F**) in corresponding areas, which was defined as a perfusion/metabolism mismatch pattern. Reduced uptake in anterior, mildly to moderately reduced uptake in lateral and inferior wall in both ^99m^Tc-MIBI SPECT (**G**) and ^18^F-FDG PET imaging (**H**), which was defined as a severe perfusion/metabolism match pattern in anterior and mild-to-moderate match pattern in lateral and inferior wall.

Detailed information about the procedures and assessment of cardiac MRI and SPECT/PET imaging can be found in Supplementary methods. In brief, an identical 17-segment model was used to divide the LV into six basal, six mid-ventricular, four distal segments, and the apex. LGE was defined as area of signal enhancement ≥2 SD of the signal of non-enhanced myocardium. Areas of LGE were classified into three categories: non-LGE, mid-wall LGE and transmural LGE. Segmental ^99m^Tc-MIBI and ^18^F-FDG uptake were scored using a 4-grade system (0 = normal uptake, 1 = mildly reduced uptake, 2 = moderately reduced uptake, 3 = severely reduced uptake). Segments were divided into four groups with different patterns of perfusion/ metabolism: normal perfusion/metabolism, perfusion/metabolism mismatch, mild-to-moderate perfusion/metabolism match, and severe perfusion/metabolism match. Representative examples of LGE-positive and perfusion/metabolism abnormality images can be found in Fig. 1.

### Follow-up and points

Follow-up started at inception with thyroid function testing. We contacted patients by phone every six months according to our follow-up schedule. Patients’ medical records would be examined if they were re-admitted into hospital. Median duration of follow-up was 36 months (1 to 58 months). Endpoints of the study were cardiac death and all-cause death. Cardiac death was defined by the occurrence of sudden death without an autopsy, cardiac arrest, or death attributable to significant arrhythmia, progressive heart failure. All-cause deaths were considered as all deaths from any natural cause. Patients lost to follow-up were censored upon last contact with them.

### Statistical analysis

Statistical analysis was assessed with SPSS statistical package for Windows 18.0. All continuous variables are presented as means ± SD and analysis of variance was used to compare means across multiple groups. Non-continuous and categorical variables are presented as frequencies or percentages and were compared using the χ^2^ test. Cochran-Armitage trend test was used to explore the distribution trend of myocardial injuries across FT3 quartiles. To adjust for the traditional risk factors, multivariable logistic regression analysis was performed while evaluating the relationship between LGE, myocardial perfusion/metabolic parameters and thyroid hormone levels. Cox proportional hazards model was used to estimate hazard ratio (HR). Kaplan-Meier analysis was used to study cumulative survival of different groups. A P-value less than 0.05 was considered statistically significant.

## Results

### Baseline clinical characteristics by quartiles of FT3 level

Table 1 shows the baseline clinical characteristics and thyroid hormone levels of the study population. Patients with IDCM were divided into four groups by quartiles of FT3 level (<2.53, n = 20; 2.53–2.76, n = 16; 2.77–3.19, n = 18; >3.19, n = 17). The quartile 1 group had the highest percentage of female and the lowest percentage of smokers. Follow-up duration for the first 3 groups was shorter than that in the quartile 4 group. Significant, differences were also detected for LVEF, TSH, FT3, and FT4. Patients in quartile 1 group, in which lowest fT3 level was present, had higher levels of NT-proBNP. A significantly decreasing trend could be detected with respect to LVEF and the presence of LGE across the listed FT3 quartiles. No significant difference was found with regard to age, blood pressure, comorbidities and medications.

### The comparison of myocardial perfusion/metabolism patterns and LGE segments

With the semi-quantitative 17-segments model, a total of 1207 segments were analyzed in the present study and detailed distribution of the segment types and percentages are shown in Table 2. From quartile 1 to 4, significant decreasing trend could be detected with respect to the percentage of segments with LGE (23.53%, 16.54%, 5.22%, 3.11%, respectively; P < 0.001) and perfusion abnormalities (20.88%, 16.54%, 14.05%, 9.69%, respectively; P < 0.001). As for segments with metabolic abnormalities, the lowest percentage was detected in the quartile 3 group, while those in quartile 1 and 2 were still much higher than quartile 3 (8.82%, 7.35% vs 1.63%; P = 0.032). An exception happened in quartile 4, with a slightly higher percentage but no statistical significance (4.84% vs 1.63%; P = 0.83). Further comparison was taken between different types of LGE (mid-wall or trans-mural) and patterns of perfusion/metabolism (normal, mismatch, mild-moderate match, severe match). Decreasing percentages could still be observed across the FT3 quartile, but no statistical significance was detected with respect of trans-mural LGE, which may be due to the low incidence. Segments of normal perfusion/metabolism pattern were more frequent in higher FT3 level groups, with total percentage of perfusion/metabolism abnormalities lower in these groups. However, no significant trend could be found if we compared the mismatch, mild-moderate match and severe match separately.

We also compared FT4 and TSH levels (shown in Supplementary Table S1). No significant trend could be detected between FT4 and any kind of myocardial injuries. As for TSH, an interesting finding is that patients with low TSH, classified as having subclinical hyperthyroidism, were more likely to be LGE-free and had a higher percentage of normal perfusion/metabolism.

### Risk factors for myocardial LEG and perfusion/metabolism abnormalities by logistic analysis

To establish the relationship between thyroid hormones, LGE and perfusion/metabolism abnormalities at patient level, univariate and multivariate logistic analyses were used to detect potential bias and adjust for traditional risk factors (Table 3). We applied logarithmic transformation to TSH values to achieve a normal distribution, thus converting it into log-TSH. For presence of LGE (patients with at least one segment of LGE), FT3 was the only significant risk factor in the univariate model (OR: 0.180, 95% CI: 0.059–0.550). In a multivariate model using stepwise strategy, FT3 was still significantly associated with the presence of LGE (OR: 0.140, 95% CI: 0.035–0.567), while age and diabetes mellitus were other risk factors (OR: 0.914, 95% CI: 0.854–0.978; OR: 5.925, 95% CI: 1.005–14.944). For presence of perfusion abnormalities (patients with at least one segment of perfusion abnormalities), OR value of FT3 was 0.38 in a univariate model (95% CI: 0.146–0.991) and 0.172 in multivariate model (95% CI: 0.040–0.738). Of note, renal dysfunction was also an independent risk factor for perfusion abnormalities. For presence of metabolism abnormalities (patients with at least one segment of metabolism abnormalities), FT3 was the only independent predictor in a multivariate model (OR: 0.338, 95% CI: 0.126–0.910). Both TSH and FT4 showed no significant relation with the presence of LGE and perfusion/metabolism.

### Prognostic value of the combination of LGE and FT3 level

**Figure 2:**
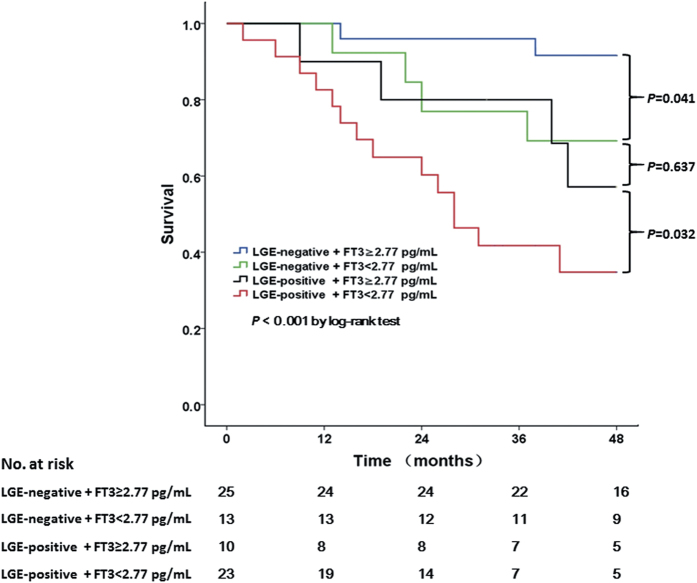
Kaplan-Meier curves comparing the probability of all-cause death. The LGE-positive+ FT3 < 2.77 pg/mL group had the worst prognosis. Importantly, survival in the LGE-negative+ FT3 < 2.77 pg/mL group was intermediate, but comparable with that in the LGE-positive+ FT3 ≥ 2.77 pg/mL group (p = 0.637).

During follow-up, there were 23 cumulative deaths, of which 19 were determined as cardiac. Kaplan-Meier plot is shown in Fig. 2. In univariate Cox survival analysis (shown in Supplementary Table S2), the presence of LGE and FT3 < 2.77 pg/mL were significantly associated with all-cause mortality, with the presence of perfusion/metabolism abnormalities having no impact. A multivariate Cox model was then performed with 3 different models shown in Table 4. In model 1, factors that were significant in the univariate analysis were put in to establish risk factors for prognosis. Both LGE-positive (HR: 5.489, 95% CI: 1.451–20.76) and FT3 < 2.77 pg/mL (HR: 2.181, 95% CI: 0.627–7.589) showed independent association with worse outcome in model 1. Further, we combined FT3 < 2.77 pg/mL and LGE into 4 classification in model 2: LGE-negative+ FT3 ≥ 2.77 pg/mL (as reference); LGE-negtive+ FT3 < 2.77 pg/mL; LGE-positive+ FT3 ≥ 2.77 pg/mL; LGE-positive+ FT3 < 2.77 pg/mL. Other factors in model 1 were still kept. In model 2, LGE-positive+ FT3 ≥ 2.77 pg/mL (HR: 5.490, 95% CI: 2.645–9.823) and LGE-positive+ FT3 < 2.77 pg/mL (HR: 7.908, 95% CI: 2.433–14.908) were significantly associated with all-cause mortality. Finally, the best predictive model (model 3) adjusted for significant predictors selected by a stepwise method based on Models 1 and 2 was performed. Again, LGE-positive+ FT3 ≥ 2.77 pg/mL (HR: 4.966, 1.851–8.658) and LGE-positive+ FT3 < 2.77 pg/mL (HR: 8.623, 95% CI: 3.626–16.438) were independent predictors for prognosis. Again, TT3 and TT4 showed no predicting value.

## Discussion

Our study explored the relationship between thyroid status and myocardial injury evaluated by cardiac MRI and ^99m^Tc-MIBI SPECT/^18^F-FDG PET imaging in patients with IDCM. The results indicated a significantly decreasing trend of myocardial fibrosis and perfusion/metabolism abnormalities across the quartiles of FT3. After adjusting for conventional risk factors, low FT3 level was still independently associated with the presence of LGE, perfusion abnormalities and metabolism abnormalities. In survival analysis, the combination of LGE-positive and FT3 < 2.77 pg/mL was identified as the strongest predictor of all-cause death. The present study confirmed and extended previous knowledge about the effects of thyroid hormone levels on IDCM.

One of the major findings in our study was the significantly decreasing trend of LGE-positive segments across FT3 quartiles. Histological basis for LGE in DCM is myocardial replacement fibrosis, which refers to reparative microscopic scarring that follows myocyte death^20^. That is to say, low thyroid function may contribute to myocardial fibrosis in IDCM. As early as 1992^14^, Yao J *et al*. first reported that TH-induced myocardial hypertrophy could be distinguished by decreased biosynthesis of type I collagen at the level of mRNA and protein. An *in vitro* experiment by Chen WJ *et al*. demonstrated that hypothyroidism can lead to increased abundance of mRNA for pro-α1(I) collagen and T3-dependent repression was through thyroid hormone receptor (TR) β1^21^. In addition, THs could accelerate the breakdown of collagen types I and III by increased matrix metalloproteinase-1 activity from suppression of endogenous tissue inhibitors of metalloproteinase^13^. Results obtained from our study are consistent with the above findings from animal experiments which revealed the up-regulation of collagen gene expression in relatively low TH conditions.

In an UK cohort of 472 non-ischemic DCM patients, those with LGE exhibited a greater degree of left ventricular dilatation and systolic impairment and were re-classified into higher risk for all-cause mortality^16^. A recently published meta-analysis also demonstrated that LGE in DCM was associated with increased risk of poor prognosis and might help guide risk stratification^17^. In the present study, both low FT3 level and presence of LGE were independent predictor for mortality and the combination of FT3 level and LGE provides more information for assessing the prognosis of IDCM. These findings, together with previous studies indicating the adverse effects of low thyroid function on DCM, suggest that TH levels should be taken as an important prognostic factor. In addition, animal studies have indicated that TH treatment may inhibit or even reverse myocardial fibrosis in heart diseases, implicating the presence and extent of LGE may act as an index in TH treatment for DCM in the future^22^,^23^.

Another interesting finding in our study is that low FT3 level contributed to impaired myocardial blood flow. Regional myocardial ischemia in IDCM has been determined from some studies^24^,^25^ and described typically as a perfusion/metabolism mismatch area in our previous study^18^. Blood flow impairment in DCM appears to be at the microvascular level rather than with blockage of large coronary vessels. In non-cardiac patients with subclinical hypothyroidism, impaired coronary blood flow that is reversible with TH treatment has been shown^26^. Pertinent to these findings, our previous studies with animal models demonstrated that hypothyroidism led to impaired myocardial blood flow due to a dramatic loss of arterioles, with TH treatment restoring myocardial flow in DCM and preventing subsequent myocyte loss and replacement fibrosis^7^. Akt/PI3K/NO signaling has been suggested to be involved in TH-induced vasodilation, which can be observed within a matter of minutes after incubating *in vitro*^11^. T3 can also induce sprouting angiogenesis in adult myocardium of hypothyroid mice *in vitro* through a PDGF-Akt-dependent signal^22^.

In the present study, patients with low FT3 level tended to be at higher risk of myocardial metabolic abnormalities. Bengel FM and colleagues reported that cardiac oxygen consumption was reduced in patients with hypothyroidism and the reduction was associated with reduced contractility^27^. There is speculation that the myocardium senses hypoxia in various heart diseases and is programmed to activate type 3 iodothyronine deiodinase (DIO3) which converts T4/T3 to inactive forms of THs, lowering tissue T3 levels in response^28^. However, Bengel FM *et al*. found that estimates of cardiac work are more severely suppressed than those of oxidative metabolism, suggesting decreased myocardial efficiency in hypothyroidism. This is pertinent to our previous animal studies suggesting the hypothyroid heart is inefficient^7^. A recent study used the TH analogue, diiodothyropropionic acid (DITPA), to treat HF patients^29^. Although cardiovascular benefits were noted, DITPA was not well tolerated and treated patients had increased heart rate and weight loss, suggesting the dose was too high. As myocardial metabolic status changes directly and rapidly with TH levels and this relationship can either be a reflection of therapeutic effects or sign of adverse effects, it provides an index for dosage titration. Inhibition or reversal of fibrosis and improved myocardial blood flow may provide long-term benefits from TH treatment in HF, while myocardial metabolism may provide rapid feedback on treatment effects.

Although no significant association was found between other thyroid parameters and myocardial injuries, patients with TSH below the reference range should be noted. In this subclinical hyperthyroid group, the percentage of LGE-positive segments was significantly lower. This finding corresponds to an interesting result of our previous study in which the prevalence of atrial fibrillation was unexpectedly lower in the subclinical hyperthyroid group than in other thyroid states^4^. At that time, a possible explanation was given that usage of beta-blockers in our cohort was more frequent, thus blunting the potential pathophysiological effects of subclinical hyperthyroidism. Currently, we have a more reasonable implication to explain this result which is inconsistent with the population-based cohort^30^. Although the degree of fibrosis was only evaluated in the left ventricle in the present IDCM cohort, it is reasonable to speculate that fibrosis likely extended to atria. The unexpected low percentage of LGE in subclinical hyperthyroid also implicates that restoration of TH level should be taken into account in treatment for HF patients in the future.

Despite the encouraging findings, our study has some limitations. The first one is that multiple thyroid tests were only available in 25% of the initially evaluated patients, so the present study could not resolve the ambiguity due to a transient change of thyroid status which is frequent in exacerbated HF. Second, thyroid function profiles were evaluated according to serum TH level rather than myocardial tissue level. As mentioned before, myocardial T3 could be down-regulated by the induced DIO3 in HF, which is independent from changes of serum TH level. Finally, because of the limited population in our study, the role of TH levels and myocardial injuries in IDCM survival needs to be further evaluated in a large population. However, strengths of the present study include the well-characterized IDCM cohort, complete information of cardiac MRI and radionuclide tests, and the exclusion of drug administration that might affect thyroid profile.

## Conclusion

We found a clear relationship between FT3 level and myocardial injuries detected by semi-quantitative evaluation of myocardial fibrosis and ischemic/viable myocardium. Combining cardiac MRI and ^99m^Tc-MIBI SPECT/^18^F-FDG PET imaging, the present study confirmed and extended previous knowledge based on animal experiments about the effects of thyroid hormone levels on IDCM, providing physicians with more comprehensive information with implications for TH therapeutic efficiency in HF.

## Additional Information

**How to cite this article**: Wang, W. *et al*. Free Triiodothyronine Level Correlates with Myocardial Injury and Prognosis in Idiopathic Dilated Cardiomyopathy: Evidence from Cardiac MRI and SPECT/PET Imaging. *Sci. Rep.* **6**, 39811; doi: 10.1038/srep39811 (2016).

**Publisher's note:** Springer Nature remains neutral with regard to jurisdictional claims in published maps and institutional affiliations.

## Supplementary Material

Supplementary Files

## Figures and Tables

**Table 1:** Baseline Characteristics of Study Population by Quartiles of FT3 Level.

	Quartiles of FT3 level (pg/mL)				
	<2.53n = 20	2.53–2.76n = 16	2.77–3.19n = 18	>3.19n = 17	*P* value
Age (year)	57 (46–63)	48 (36–60)	56 (49–66)	54 (49–59)	0.112
Female (n, %)	10 (50.0%)	6 (37.5%)	5 (27.8%)	3 (17.6%)	0.017
Smoking, (n, %)	4 (20.0%)	6 (37.5%)	7 (38.9%)	9 (52.9%)	0.024
BMI (kg/m^2^)	24.2 (3.79)	24.91 (5.89)	24.63 (1.7)	25.87 (2.92)	0.635
Blood pressure					
Systolic (mmHg)	112 (18)	107 (18)	119 (19)	117 (14)	0.231
Diastolic (mmHg)	70 (9)	71 (11)	71 (11)	76 (11)	0.268
NYHA III–IV class, (n, %)	18 (90.0%)	11 (68.7%	13 (72.2%)	13 (76.5%)	0.411
Comorbidities (n, %)					
Atrial fibrillation	4 (20.0%)	4 (25.0%)	2 (11.1%)	2 (11.8%)	0.026
Diabetes mellitus	5 (25.0%)	2 (12.5%)	3 (16.7%)	1 (5.9%)	0.439
Dyslipidemia	7 (35.0%)	6 (37.5%)	5 (27.8%)	6 (35.3%)	0.924
Anemia	3 (15.0%)	4 (25.0%)	2 (11.1%)	1 (5.9%)	0.473
Renal dysfunction	4 (20.0%)	5 (31.3%)	4 (22.2%)	2 (11.8%)	0.592
Medications, (n, %)					
ACEi/ARB	11 (55.0%)	10 (62.5%)	15 (83.3%)	12 (70.6%)	0.291
Beta blocker	18 (90.0%)	13 (81.3%)	18 (100.0%)	12 (70.6%)	0.078
Aldosterone antagonists	13 (65.0%)	8 (50.0%)	11 (61.1%)	6 (35.3%)	0.498
Diuretics	19 (95.0%)	15 (93.8%)	17 (94.4%)	14 (82.4%)	0.482
Cardiac MRI measurements					
LVEF (%)	23 (6)	24 (8)	28 (9)	28 (7)	0.009
LVEDV index (mL/m^2^)	170.5 (72.1)	162.2 (70.5)	145.3 (61.7)	132.5 (50.6)	0.032
LVESV index (mL/m^2^)	137.1 (56.2)	120.6 (60.4)	129.3 (49.1)	105.7 (53.5)	0.073
LV mass (g)	142 (7)	146 (9)	149 (7)	147 (8)	0.154
Presence of LGE	13 (65.0%)	10 (62.5%)	7 (38.8%)	3 (17.6%)	0.015
Presence of perfusion abnormality	14 (70.0%)	9 (56.2%)	13 (72.2%)	6 (35.3%)	0.098
Presence of metabolism abnormality	10 (50.0%)	7 (43.8%)	3 (16.7%)	3 (17.6%)	0.058
TSH (mIU/L)	1.55 (0.89–3.5)	2.32 (1.44–2.93)	2.19 (1.52–3.1)	0.86 (0.6–1.43)	0.444
FT4 (ng/dlL)	1.25 (1.05–1.39)	1.22 (1.13–1.27)	1.21 (1.10–1.43)	1.27 (1.09–1.43)	0.291
FT3 (pg/mL)	2.29 (1.92–2.46)	2.65 (2.60–2.70)	3.01 (2.88–3.13)	3.38 (3.24–3.61)	<0.001

Abbreviation: BMI, body mass index; NYHA, New York Heart Association; ACEi, angiotensin-converting enzyme inhibitor; ARB, angiotensin receptor blocker; LV, left ventricle; EF, ejection fraction; EDV, end-diastolic volume; ESV, end-systolic volume; LGE, late gadolinium enhancement; TSH, thyroid stimulating hormone; FT4, free thyroxin; FT3, free triiodothyronine. The data are presented as n (%), median (first quartile, third quartile) or mean (SD).

**Table 2:** Segments Analysis of LGE and Myocardial Perfusion/Metabolism Patterns by Quartiles of FT3 level.

	Quartiles of FT3 level (pg/mL)				
	<2.53 (No. of segments: 340)	2.53–2.76 (No. of segments: 272)	2.77–3.19 (No. of segments: 306)	>3.19 (No. of segments: 289)	*P* for trend^*^
Cardiac MRI measurements (No. of segments, %)					
Total segments with LGE	80 (23.53%)	45 (16.54%)	16 (5.22%)	9 (3.11%)	<0.001
Mid-wall	55 (16.18%)	22 (8.09%)	14 (4.58%)	8 (2.77%)	<0.001
Trans-murual	25 (7.35%)	23 (8.46%)	2 (0.65%)	1 (0.35%)	<0.001
^99m^Tc-MIBI SPECT/^18^F-FDG PET imaging (No. of segments, %)					
Segments with perfusion abnormalities	71 (20.88%)	45 (16.54%)	43 (14.05%)	28 (9.69%)	<0.001
Segments with metabolism abnormalities	30 (8.82%)	20 (7.35%)	5 (1.63%)	14 (4.84%)	0.022
Perfusion/metabolism match pattern					
Normal	269 (79.12%)	227 (83.46%)	264 (86.27%)	261 (90.31%)	<0.001
Mismatch	41 (12.06%)	25 (9.19%)	38 (12.42%)	14 (4.84%)	0.016
Mild-moderate match	22 (6.47%)	12 (4.41%)	2 (0.65%)	7 (2.42%)	0.005
Severe match	8 (2.35%)	8 (2.94%)	3 (0.98%)	7 (2.42%)	0.635

^*^P value for trend estimated by using Cochran-Armitage trend test between FT3 quartiles and percentage of LGE.

**Table 3:** Predictors of LGE and Perfusion/Metabolism Abnormalities by Logistic Regression.

	Presence of LGE			Presence of perfusion abnormalities			Presence of metabolism abnormalities		
	OR	95% CI	*P* value	OR	95% CI	*P* value	OR	95% CI	*P* value
Univariate analysis									
FT3	0.180	0.059–0.550	0.003	0.380	0.146–0.991	0.048	0.338	0.126–0.910	0.032
FT4	0.373	0.050–2.759	0.334	0.432	0.068–2.729	0.372	0.644	0.088–4.709	0.665
Log-TSH	0.982	0.930–1.038	0.524	1.067	0.67–1.699	0.785	1.073	0.661–1.743	0.774
Age	0.972	0.934–1.012	0.165	0.963	0.922–1.005	0.085	0.981	0.942–1.022	0.353
Male	0.808	0.302–2.164	0.671	1.053	0.388–2.858	0.920	0.707	0.251–1.993	0.512
LVEF	0.989	0.950–1.030	0.609	0.973	0.932–1.014	0.194	0.983	0.941–1.028	0.451
BMI	0.906	0.787–1.044	0.174	0.975	0.858–1.107	0.693	0.866	0.737–1.019	0.083
Diabetes	2.288	0.605–8.656	0.223	8.750	1.053–12.699	0.045	0.413	0.082–2.088	0.285
Smoking	0.767	0.290–2.029	0.593	1.169	0.436–3.137	0.756	0.889	0.315–2.510	0.824
Hypertension	0.481	0.175–1.321	0.156	0.500	0.184–1.361	0.175	0.242	0.071–0.828	0.024
Anemia	0.365	0.036–3.685	0.393	0.211	0.021–2.142	0.188	0.682	0.067–6.937	0.746
Renal dysfunction	0.500	0.151–1.651	0.255	0.257	0.077–0.859	0.027	0.450	0.113–1.785	0.256
Dyslipidemia	0.779	0.288–2.111	0.624	0.693	0.254–1.886	0.473	0.409	0.130–1.292	0.128
Multivariate analysis*									
FT3	0.140	0.035–0.567	0.006	0.172	0.040–0.738	0.018	0.281	0.081–0.971	0.045
Age	0.914	0.854–0.978	0.009	0.946	0.891–1.004	0.070			
Diabetes	5.925	1.005–14.944	0.049	9.504	0.922–17.957	0.059			
Renal dysfunction				0.101	0.016–0.648	0.016			
Hypertension							0.455	0.11–1.876	0.276

^*^Significant predictors selected by univariate analysis were just put in. Stepwise strategy was used for other factors, in which predictors with P value < 0.1 were kept.

**Table 4:** Multivariate Cox Survival Analysis Based on FT3 Level and Presence of LGE.

	Model 1^*^			Model 2^†^			Model 3^‡^		
	HR	95% CI	*P* value	HR	95% CI	*P* value	HR	95% CI	*P* value
Age (per 5 years)	1.073	1.056–1.983	0.040	1.094	1.059–1.351	0.043	1.090	1.012–1.341	0.035
Gender (male)	1.629	0.968–2.217	0.091	1.492	0.453–2.381	0.136			
LVEF (per 5%)	0.754	0.613–0.992	0.026	0.781	0.539–0.974	0.035	0.746	0.609–0.935	0.029
Anemia	3.712	1.203–10.020	0.049	3.519	1.197–8.356	0.022			
Renal Dysfunction	2.642	1.136–4.184	0.039	2.216	0.972–3.923	0.065			
FT3 < 2.77	2.181	1.627–7.589	0.020						
Presence of LGE	5.489	1.451–10.76	0.012						
LGE-negtive+ FT3 < 2.77				2.127	0.830–5.107	0.145			
LGE-positive+ FT3 ≥ 2.77				5.490	2.645–9.823	0.011	4.966	1.851–8.658	0.007
LGE-positive+ FT3 < 2.77				7.908	2.433–14.908	0.002	8.623	3.626–16.438	0.001

^*^Multivariate Cox model selected by a stepwise method with factors that were significant in the univariate analysis and established risk factors for prognosis (age, gender, NYHA classification).

^†^Model with the combination of LGE and FT3 < 2.77, adjusted for predictors selected by Model 1.

^‡^Best predictive model in which predictors with P value < 0.1 were kept, adjusted for significant predictors selected by a stepwise Cox regression analysis based on Models 1 and 2.
