# Supplementary material for: Free Triiodothyronine Level Correlates with Myocardial Injury and Prognosis in Idiopathic Dilated Cardiomyopathy: Evidence from Cardiac MRI and SPECT/PET Imaging
Source: Sci Rep. 2016 Dec 22;6:39811. doi: 10.1038/srep39811 (PMC5177909; doi:10.1038/srep39811)
Supplement: Supplementary Files [file srep39811-s1.pdf]

Free Triiodothyronine Level Correlates with Myocardial Injury and Prognosis in Idiopathic Dilated Cardiomyopathy: Evidence from Cardiac MRI and SPECT/PET Imaging

Wenyao Wang[1], Haixia Guan[2], Wei Fang[3], Kuo Zhang[1], A. Martin Gerdes[4], Giorgio Iervasi[5], Yi-Da Tang[1]*

[1]Department of Cardiology, State Key Laboratory of Cardiovascular Disease, Fuwai Hospital, National Center for Cardiovascular Diseases, Chinese Academy of Medical Sciences and Peking Union Medical College, Beijing, China.

[2]Department of Endocrinology and Metabolism, The First Affiliated Hospital of China Medical University, Shenyang, China.

[3]Department of Nuclear Medicine, State Key Laboratory of Cardiovascular Disease, Fuwai Hospital, National Center for Cardiovascular Diseases, Chinese Academy of Medical Sciences and Peking Union Medical College, Beijing, China.

[4]Department of Biomedical Sciences, New York Institute of Technology-College of Osteopathic Medicine, Old Westbury, New York, USA.

[5]Clinical Physiology Institute, Consiglio Nazionale delle Ricerche (CNR), Pisa, Italy.

***Correspondence to** Yi-Da Tang (Email: tangyida@fuwaihospital.org).

Department of Cardiology, State Key Laboratory of Cardiovascular Disease, Fuwai Hospital, National Center for Cardiovascular Diseases, Chinese Academy of Medical Sciences and Peking Union Medical College. No.167 Beilishi Road, Beijing 100037, China.

Supplementary Methods

Cardiac MRI

Cardiac MRI was performed on a 1.5-T scanner (Magnetom Avanto, Siemens Medical Solutions, Erlangen, Germany). We acquired scout images from HASTE sequence for morphological analysis. Retrospective electrocardiogram (ECG) gating cine images were acquired by a true fast imaging with steady-state precession sequence. To cover the entire left ventricle (LV), we got views in the two-chamber long-axis, four-chamber long-axis, LV outflow tract, and six to eight sections in the short-axis with repeated breath-holds. At 10 minutes after bolus injection of 0.2mmol/kg body weight gadolinium diethylenetriamine pentaacetic acid, delayed enhancement T1-weighted images were acquired with a phase-sensitive inversion recovery sequence. Twenty-five phases were obtained per cardiac cycle. On the bases of vertical and horizontal long axes of LV, the true short axis was determined covering LV from base to apex.

Cardiac ^{99m}Tc-MIBI SPECT and ^{18}F-FDG PET imaging

SPECT imaging was performed at rest 60min after injection of 740MBq ^{99m}Tc-MIBI, using a dual-head SPECT camera (e.com, Siemens Healthcare) centered on the 140 KeV energy peak with a 20% symmetrical energy window. Thirty projection images were acquired over a 180-degree arc (45-degree right anterior oblique to 45-degree left posterior oblique position) at 6-degree intervals. The acquisition time was 30s at each projection. The transaxial data were reconstructed using Butterworth-filtered back projection with a cutoff frequency of 0.45 and an order of 7. Tomographic images were displayed as short-, horizontal long-, and vertical long-axis slices.

^{18}F-fluorodeoxyglucose (^{18}F-FDG) served as a tracer for assessment of viability and hibernating myocardium. After a minimum 8h overnight fast, all patients initially received 50g oral glucose load 30min before the injection of 185MBq ^{18}F-FDG. Data were acquired 60min later by PET-CT (Truepoint Biography 64, Siemens Healthcare). The acquisition time was 10min for emission (three-dimensional mode) and cardiac cycle was divided into eight equal intervals. Attenuation-corrected data were reconstructed using an iterative algorithm (OSEM, 4 iterations, 8 subsets). The matrix was 128×128 pixels and the reconstruction zoom was 2.0. Short axis slices of 3 mm slice thickness were

obtained in PET images.

Image analysis

All data was transferred to a separate workstation and was assessed by two independent readers. A third blinded reader adjudicated in cases with disagreement. An identical 17-segment model was used to divide the LV into six basal, six mid-ventricular, four distal segments, and the apex. LGE was defined as area of signal enhancement≥2 SD of the signal of non-enhanced myocardium. Areas of LGE were classified into three categories: non-LGE, mid-wall LGE and transmural LGE. Segmental ^{99m}Tc-MIBI and ^{18}F-FDG uptake were scored using a 4-grade system (0= normal uptake, 1= mildly reduced uptake, 2=moderately reduced uptake, 3=severely reduced uptake). The segments were divided into four groups with different patterns of perfusion/ metabolism: group 1 (normal perfusion/metabolism) consisted of segments with normal ^{99m}Tc-MIBI (score 0) and normal ^{18}F-FDG (score 0) uptake; group 2 (perfusion/metabolism mismatch) consisted of segments with reduced ^{99m}Tc-MIBI uptake (score 1, 2 or 3) and preserved/relatively increased ^{18}F-FDG uptake (^{18}F-FDG score < ^{99m}Tc-MIBI score); group 3 (mild-to-moderate perfusion/metabolism match) consisted of segments with a mild/moderately reduced ^{99m}Tc-MIBI uptake (score 1 or 2) and a concordant mild/moderate reduction in ^{18}F-FDG uptake (score 1 or 2); group 4 (severe perfusion/metabolism match) consisted of segments with a severe reduction in ^{99m}Tc-MIBI uptake (score 3) and a concordant reduction in ^{18}F-FDG uptake (score 3).

Supplementary Figure

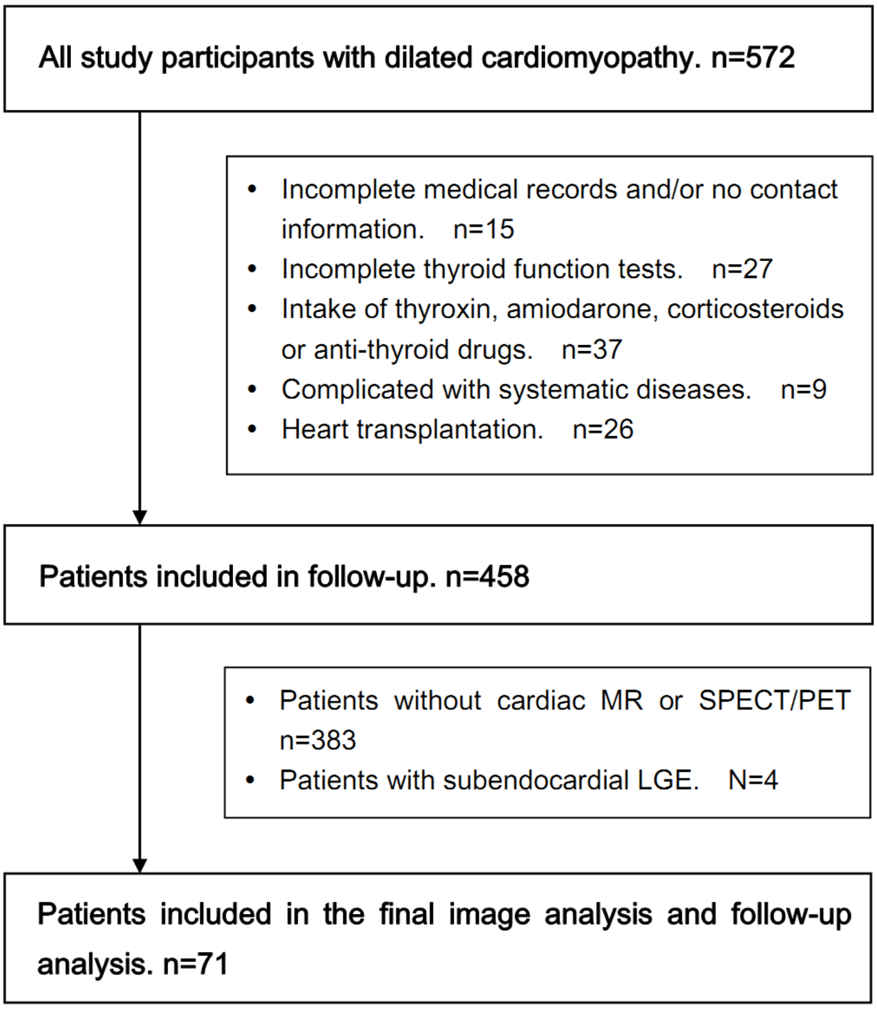

Figure S1. Study diagram showing number of patients available for baseline thyroid function test and final analysis.

Table S1. Segments Analysis of LGE and Myocardial Perfusion /Metabolism Patterns according to TSH level.

	TSH level (IU/L)				
	**< 0.55 (No. of segments: 136)**	**0.55-2.50 (No. of segments: 714)**	**2.51 – 4.78 (No. of segments: 238)**	**> 4.78 (No. of segments: 119)**	***P* for trend [a]**
Cardiac MRI measurements (No. of segments, %)					
Total segments with LGE	129 (94.85%)	631 (88.38%)	191 (80.25%)	106 (89.08%)	0.008
Mid-wall	5 (3.68%)	51 (7.14%)	25 (10.50%)	11 (9.24%)	0.027
Trans-mural	2 (1.47%)	32 (4.48%)	22 (9.24%)	2 (1.68%)	0.192
^{99m}Tc-MIBI SPECT/^{18}F-FDG PET imaging (No. of segments, %)					
Segments with perfusion abnormalities	7 (5.15%)	121 (16.95%)	39 (16.39%)	20 (16.81%)	0.052
Segments with metabolism abnormalities	1 (0.74%)	45 (6.30%)	19 (7.98%)	4 (3.36%)	0.303
Perfusion /metabolism match pattern					
Normal	129 (94.85%)	594 (83.19%)	199 (83.61%)	99 (83.19%)	0.048
Mismatch	6 (4.41%)	76 (10.64%)	20 (8.40%)	16 (13.45%)	0.118
Mild-moderate match	0 (0.00%)	28 (3.92%)	12 (5.04%)	3 (2.52%)	0.248
Severe match	1 (0.74%)	17 (2.38%)	7 (2.94%)	1 (0.84%)	0.865

[a] P value for trend estimated by using Cochran-Armitage trend test between TSH level and percentage of LGE.

Table S2. Univariate Cox Survival Analysis of Risk Factors for All-cause Mortality.

	HR	95%CI	*P* value
Age (per 5 years)	0.978	0.957-0.991	0.048
Gender (male)	1.646	0.287-2.755	0.501
BMI (per 1 kg/m^2)	1.009	0.908-1.122	0.865
NT pro-BNP (per 100 pg/mL)	1.061	1.027-1.097	<0.001
Atrial fibrillation	0.630	0.261-1.519	0.303
Diabetes mellitus	1.238	0.032-1.763	0.160
Anemia	3.488	1.004-12.116	0.049
Renal dysfunction	2.668	1.228-4.956	0.032
Thyroid hormone levels			
FT3 (per 1 pg/mL)	0.244	0.110-0.539	<0.001
FT4 (per 1 ng/dL)	0.560	0.097-3.221	0.516
TT3 (per 1 ng/mL)	0.501	0.101-2.491	0.634
TT4 (per 1 ug/dL)	1.038	0.816-1.321	0.759
TSH (per 1 mIU/L)	0.813	0.562-1.178	0.275
Cardiac MRI measurements			
LGE-positive	4.840	1.912-12.248	0.001
LVEF (per 5%)	0.824	0.698-0.972	0.021
EDV (per 10 mL)	1.013	1.000-1.046	0.078
ESV (per 10 mL)	1.026	1.000-1.097	0.092
^{99m}Tc-MIBI SPECT/^{18}F-FDG PET imaging			
Perfusion abnormalities	1.246	0.545-2.848	0.602
Metabolism abnormalities	2.043	0.915-4.563	0.081
